# Transcriptome Analysis of Ramie (*Boehmeria nivea* L. Gaud.) in Response to Ramie Moth (*Cocytodes coerulea* Guenée) Infestation

**DOI:** 10.1155/2016/3702789

**Published:** 2016-02-29

**Authors:** Liangbin Zeng, Airong Shen, Jia Chen, Zhun Yan, Touming Liu, Zhaodong Xue, Yongting Yu

**Affiliations:** ^1^Institute of Bast Fiber Crops, Chinese Academy of Agricultural Sciences, Changsha, Hunan 410205, China; ^2^Hunan Academy of Forestry Science, Changsha, Hunan 410004, China

## Abstract

The ramie moth* Cocytodes coerulea *Guenée (RM) is an economically important pest that seriously impairs the yield of ramie, an important natural fiber crop. The molecular mechanisms that underlie the ramie-pest interactions are unclear up to date. Therefore, a transcriptome profiling analysis would aid in understanding the ramie defense mechanisms against RM. In this study, we first constructed two cDNA libraries derived from RM-challenged (CH) and unchallenged (CK) ramie leaves. The subsequent sequencing of the CH and CK libraries yielded 40.2 and 62.8 million reads, respectively. Furthermore,* de novo* assembling of these reads generated 26,759 and 29,988 unigenes, respectively. An integrated assembly of data from these two libraries resulted in 46,533 unigenes, with an average length of 845 bp per unigene. Among these genes, 24,327 (52.28%) were functionally annotated by predicted protein function. A comparative analysis of the CK and CH transcriptome profiles revealed 1,980 differentially expressed genes (DEGs), of which 750 were upregulated and 1,230 were downregulated. A quantitative real-time PCR (qRT-PCR) analysis of 13 random selected genes confirmed the gene expression patterns that were determined by Illumina sequencing. Among the DEGs, the expression patterns of transcription factors, protease inhibitors, and antioxidant enzymes were studied. Overall, these results provide useful insights into the defense mechanism of ramie against RM.

## 1. Introduction

Ramie (*Boehmeria nivea* L. Gaud.), also called China grass, is a perennial herbaceous plant that belongs to the family Urticaceae. It is an important natural fiber crop, mainly grown in China, India, and other Southeast Asian and Pacific Rim countries [1]. Traditionally, ramie was planted as a fiber crop, and only the best fibers were harvested (constituting approximately 4% of the total dry matter). However, studies over the past decades have shown that ramie is rich in protein [2], and the shoots and leaves can be used as fodder for cattle and geese [3].

**Figure 1:**
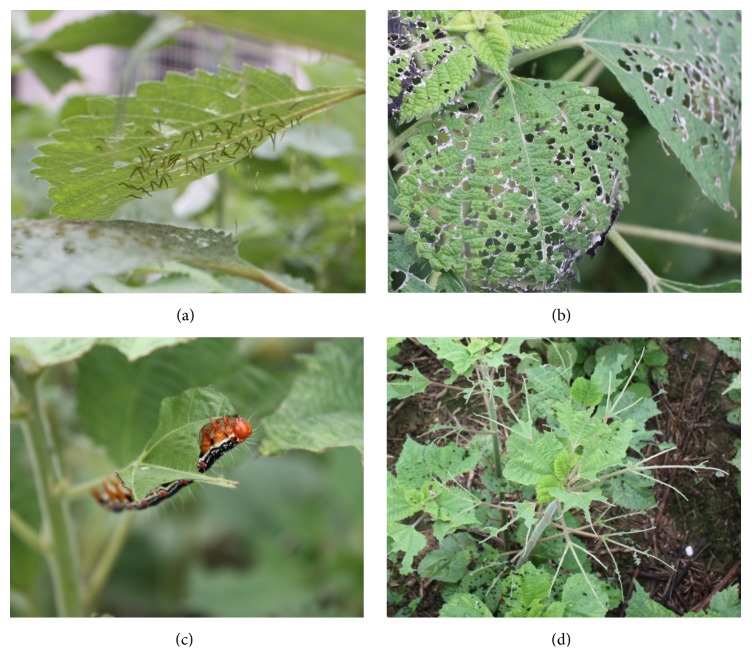
Pictures of* Cocytodes coerulea *Guenée larvae and their resulting damage to ramie leaves. (a) J2 stage larvae feeding on ramie leaves. (b) The resulting net-like structure for the ramie leaves after J2 stage herbivory of* C. coerulea*. (c) A J5 stage larva eating a ramie leaf. (d) The remaining stems and main veins after infestation of the J5 stage larvae.

*Cocytodes coerulea *Guenée, also known as the ramie moth (RM), is a destructive plant pest. It can cause severe ramie yield reductions, as it feeds on ramie leaves and new shoots [4]. This pest is widely distributed in China, Japan, India, and Southeast Asian countries. In central and south China, up to four generations per year can occur during the vegetative growth phase of ramie [5]. Female RMs lay eggs (approximately 400 eggs each) on the abaxial leaf surface of ramie, which causes the leaves to yellow. After the eggs' hatching, the RM larvae begin to feed on the leaves, which will result in a net-like pattern of damage (Figure 1), and will seriously impair the plant's photosynthetic capacity, resulting in a yield reduction.

Chemicals such as dichlorvos, Trinox, and pyrethrum ester insecticides are effective in controlling RM larvae. However, the widespread use of agrochemicals can result in serious problems referred to as “3R-problem”: residue, resistance, and resurgence. Selecting RM-resistant cultivars is therefore considered to be the most economical, effective, and environment-friendly control measure for reducing RM damage. A previous study showed that different ramie cultivars displayed varied levels of resistance to RM, which were correlated to the phenol, tannin, and oxadate contents in the leaves [6]. However, the underlying molecular mechanism of this resistance to RM is unclear.

Methods such as suppression subtractive hybridization, cDNA-amplified fragment length polymorphism (cDNA-AFLP), and microarrays have been used to detect differentially expressed genes (DEGs) and hence elucidate the molecular mechanisms of plant responses to stress [7–11]. However, these methods are time consuming. Recently, high-throughput sequencing (Illumina HiSeq, Roche/454, and ABI SOLiD platform) has become a highly effective tool to identify DEGs, owing to its speed, cost-efficiency, and high-throughput ability. For example, high-throughput sequencing was employed to study the transcriptome profiles of* Gossypium hirsutum* [12, 13] and* Barbarea vulgaris* [14] after infestation with herbivorous larvae or insects.* B. vulgaris* can resist the diamond back moth (DBM) and other insects by producing feeding-deterrent triterpenoid saponins. By investigating the DBM-induced changes in the transcriptome, triterpenoid saponin biosynthetic pathways and regulatory networks were analyzed, and the genes involved in these pathways were consequently identified [14]. Furthermore, by comparing transcriptome changes of cotton before and after aphid and whitefly infestation, the expression of some marker genes involved in phytohormonal-mediated plant resistance was found to be suppressed after insect infestation [12]. The suppressed marker genes included cationic peroxidase 3, lipoxygenase I, and nonspecific lipase, which suggest that insects suppress plant resistance in order to facilitate their feeding. In this study, we used Illumina sequencing to compare the levels of differentially expressed genes in leaves of ramie plants with and without RM infestation. The underlying mechanisms involved in ramie defense against insects are subsequently discussed.

## 2. Materials and Methods

### 2.1. Plant Growth, Pest Inoculation, and RNA Preparation

Ramie Chuanzhu 8, which is moderately resistant to RM, was used in this study. Ramie seedlings were prepared with the shoot-cutting propagation method. The seedlings were cultured in a climate chamber at 26°C  ±  1°C, 75%  ±  1% relative humidity, with a photoperiod of 14 : 10 (L : D). RM egg masses attached to the back of ramie leaves were collected from infested ramie fields at the Institute of Bast Fiber Crops (112.11′E, 28.51′N). For hatching, the eggs were subjected to the same conditions as the ramie seedlings, and J2 larvae were used as inoculums. To prepare the challenged plants (CH), two J2 larvae were transferred to the ramie seedlings onto the fourth leaf from the shoot apex. To stimulate their appetite, larvae were kept on fasting for 12 h prior to inoculation. At 12, 24, 48, and 72 h after inoculation, the two topmost undamaged leaves were sampled. Unchallenged control (CK) plants were sampled simultaneously. There were five plants per treatment, and their sampled leaves were pooled per treatment. Overall, eight pooled samples were obtained (CK12, CK24, CK48, CK72, CH12, CH24, CH48, and CH72). Total RNA from each pooled sample was extracted using a commercially available EASYspin plus Total RNA kit (Aidlab, Beijing, China), following the manufacturer's protocol. The obtained RNA was subsequently stored at −80°C.

### 2.2. Transcriptome Library Preparation and Sequencing

For transcriptome sequencing of the CK and CH samples, equal amounts of extracted RNA from all four sampling time points were mixed. From these two mixtures, 5 *μ*g mixed RNA was used to construct two sequencing libraries, each corresponding to either the CK or the CH sample. From the mixed RNA samples, mRNA was purified by briefly allowing it to bind to magnetic oligo (dT) beads, and it was subsequently broken into short fragments by the addition of a fragmentation buffer (Ambion). The short mRNA fragments were used as templates for synthesizing first-strand cDNA with a random hexamer-primer, dNTPs, RNase H, DNA polymerase I, and GEX. A second-strand buffer was then added to synthesize second-strand cDNA. The resulting cDNA fragments were purified with a QiaQuick PCR extraction kit and subsequently eluted in EB buffer from the kit for end repair and the addition of poly(A) tails. The fragments were then subjected to agarose gel electrophoresis. Suitable fragments were recovered and used as templates for PCR amplification. Finally, the amplified library was sequenced using an Illumina HiSeq*™* 2000 platform at Biomarker Technologies Co., LTD, Beijing, China. The resulting data (i.e., the clean reads, counts, and RPKM values) have been submitted to the Gene Expression Omnibus (GEO) at the NCBI, with the GEO accession number GSE66447.

### 2.3. Assembly and Functional Annotation

In order to yield clean reads, adaptor-only reads, reads containing more than 5% unknown nucleotides, and low-quality reads (reads containing more than 50% of bases with a* Q*-value of ≤20%) were filtered out of the results after sequencing. To generate nonredundant unigenes, clean reads were subsequently assembled* de novo,* using the Trinity method with an optimized* K*-mer length of 25 [15].

For annotation, the unigenes were first searched against the NCBI nonredundant (Nr) database (http://www.ncbi.nlm.nih.gov/), the Swiss-Prot database (http://www.expasy.ch/sprot), the Clusters of Orthologous Groups (COG) database (http://www.ncbi.nlm.nih.gov/COG/), the Gene Ontology (GO) database (http://geneontology.org/), and the Kyoto Encyclopedia of Genes and Genomes (KEGG) protein database (http://www.genome.jp/kegg) using local BLASTx (with an *E* value < 10^−5^) to obtain homologous protein information. With Nr annotation, we used the Blast2GO program [16] to obtain GO annotation according to the molecular function, biological process, and cellular component ontology. WEGO software [17] was subsequently used to obtain GO functional classification of all unigenes. In addition, all unigene sequences were aligned to the COG database to predict and classify their possible functions.

### 2.4. Identification of DEGs

The transcript level of each expressed gene was calculated and normalized to reads per kilobase of exon model per million mapped read (RPKM) [18]. DESeq software (http://www-huber.embl.de/users/anders/DESeq/) [19] was used to find differentially expressed genes using pairwise comparisons, and the results of all statistical tests were corrected for multiple testing with the Benjamini-Hochberg false discovery rate (FDR) of *P* < 0.01. Sequences were regarded to be significantly differentially expressed if the adjusted *P* value was <0.01, and the absolute value of the log_2_ (fold change) was ≥1. Here, the fold change was calculated using the RPKM value of the CH library divided by that of the CK library.

### 2.5. Pathways Enrichment of DEGs

Pathway enrichment analysis based on the KEGG pathway database (http://www.genome.jp/kegg) was used to identify markedly enriched metabolic pathways or signal transduction pathways in differentially expressed genes, compared with the whole genome background. The following equation was used for the calculations:

(1) $$\begin{array}{c} p=1-\overset{m-1}{\underset{i=0}{\sum }}\frac{\left(\begin{array}{c} M\\ i \end{array}\right)\left(\begin{array}{c} N-M\\ n-i \end{array}\right)}{\left(\begin{array}{c} N\\ n \end{array}\right)}, \end{array}$$

where *N* is the number of all genes with a KEGG annotation, *n* is the number of DEGs in *N*, *M* is the number of all genes annotated to specific pathways, and *m* is the number of DEGs in *M*.

### 2.6. Quantitative Real-Time PCR (qRT-PCR) Analysis

To verify genes that were differentially expressed in RM-challenged samples compared with unchallenged ones, qRT-PCR was performed, using an iQ SYBR Green Super Mix kit (Bio-Rad) on an iCycler iQ system (Bio-Rad, Hercules, CA, USA). Gene-specific primers of 13 candidate genes (Table S1 in Supplementary Material available online at http://dx.doi.org/10.1155/2016/3702789) were designed using the Primer Premier 5.0 software. The ramie gene encoding actin, which displays a stable expression under different stress conditions [20], was used as an internal control for data normalization. For each sample, first-strand cDNA was synthesized from 1 *μ*g of the pooled RNA sample of the CK or CH plants, using a RevertAid First-Strand cDNA Synthesis Kit (Thermo Scientific, Fermentas, Vilnius, Lithuania), according to the manufacturer's instructions. All reactions were performed in triplicate. Expression levels of each gene are presented as the fold change relative to that of the control gene, calculated with the 2^−ΔΔCt^ method [21].

## 3. Results

### 3.1. Sequencing and* De Novo* Assembly

**Figure 2:**
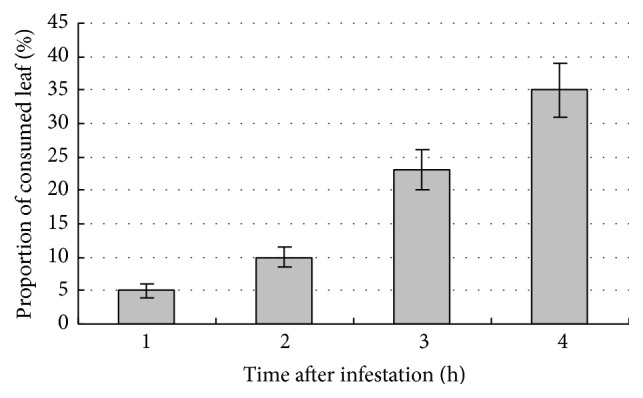
The proportion of leaf consumed per larva (J2 stage) at different time after infestation.

To study differently expressed genes in ramie leaves after RM infestation, ramie leaves were subjected to the feeding larvae of* C. coerulea *Guenée (J2). At 12, 24, 48, and 72 h after inoculation, the proportion of consumed leaves area by one larva were approximately 5%, 10%, 23%, and 35%, respectively, for each infested leaf (Figure 2). Leaves of CH (challenged) and CK (control) plants were sampled at these time points. RNA extracted from CH and CK samples was used to construct cDNA libraries with a fragment length of 200 bp. Fragments were then sequenced using Illumina paired-end sequencing technology. Raw sequencing data were obtained by base-calling transformation from the sequencing-received raw image data. After filtering, 40,187,616 and 62,784,364 clean reads of 90 bp in length were obtained for the CK and CH libraries, respectively (Table 1). The GC content of sequence data from the two libraries was 51.29% and 50.73%, respectively. Furthermore, the CycleQ20% was 100% for both, which indicates that the accuracy and quality of the sequencing data were sufficient for further analysis.

*De novo* assembly was performed using the Trinity method. Sequences that were not extended on either end were defined as being a unigene. Clean reads of CK and CH libraries were assembled into 26,759 (mean length 1,037) and 29,988 unigenes (mean length 982), respectively. To obtain integrated information, reads of the two libraries were merged as well. Finally, 46,533 unigenes for ramie leaves (Chuanzhu 8) were obtained, with an average unigene length of 845 bp (Table 1, Figure S1).

### 3.2. Functional Annotation and Classification

All of the unigenes assembled were aligned to five public protein databases (Table 2). For these databases, the alignment resulted in 24,217 homologues (52.04% of all unigenes) with the Nr database, 17,948 (38.57%) with Swiss-Prot, 20,736 (44.56%) with GO, 9,089 (19.53%) with COG, and 5,929 (12.74%) with KEGG. Overall, 24,327 (52.27%) unigenes were functionally annotated, and 11,842 of them were larger than 1,000 bp in length. Approximately half of all unigenes (22,206, 47.73%) had no database hits, suggesting that these genes are novel to the databases or specific for ramie.

Based on the functional annotation information, 20,736 unigenes were categorized to 56 different GO terms that followed three main ontology groups: biological process, cellular component, and molecular function (Figure S2). Because of the matched homologous proteins in databases, some genes were assigned to more than one term. Overall, the 56 GO terms included 221,515 genes. Among these, 113,484 (51.23%) genes were classified into the biological process group and accounted for the greatest proportion, followed by 79,114 (35.71%) in the cellular component and 28,920 (13.06%) in the molecular function group. A total of 136 unigenes were annotated to be involved in biological processes associated with defense responses to insects (Table S2).

All unigenes were aligned to the COG database in order to predict and classify their possible functions. From the 24,217 unigenes (with Nr hits), 9,089 could be categorized according to the COG classifications (Figure S3). From the 25 categories, the cluster “general function prediction only” (2,314, 18.32%) represented the largest group, followed by “replication, recombination, and repair” (1,216, 9.62%), “translation, ribosomal structure, and biogenesis” (1,106, 8.75%), “transcription” (1,085, 8.59%), and “signal transduction mechanisms” (938, 7.42%). Collectively, 3,863 unigenes were assigned to various metabolic processes, and 144 unigenes were assigned to “defense mechanisms.” In contrast, only a few unigenes were assigned to “cell motility” (17, 0.13%) and “nuclear structure” (1, 0.01%), and none were assigned to “extracellular structures.”

### 3.3. Identification of 1980 DEGs Induced by RM Infestation

**Figure 3:**
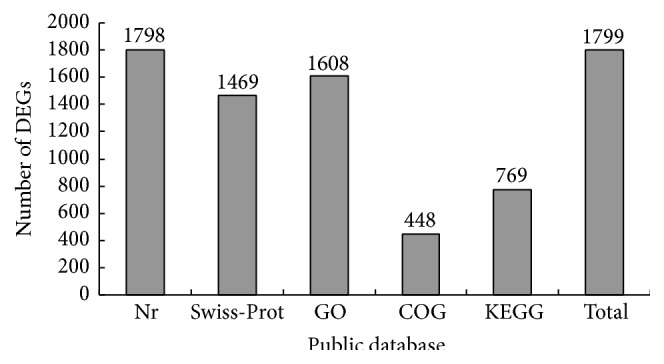
Numbers of differentially expressed genes (DEGs) annotated in five public databases.

To identify the genes involved in RM responses, the transcriptomes of RM-infested and uninfested samples were compared. The number of clean reads for each gene was calculated. Individual sets of reads were then mapped back to the previously assembled transcript and counted as a proxy for gene expression. Differences in transcript expression between the CH and CK samples were identified with an algorithm developed by Audic and Claverie [22]. As a result, 1,980 genes (750 upregulated and 1,230 downregulated) with at least a twofold difference between the CK and CH treatment were identified as DEGs. According to the annotation information of the unigenes described above, 1,799 of these DEGs were functionally annotated (Figure 3). Among these DEGs, 87 transcription factors (TFs) belonging to 22 families were found for plants infested with RM (Table 3, Table S3). Furthermore, 16 DEGs involved in the defense response to insects (by GO annotation) were identified for the RM-infested plants (Table S2).

### 3.4. Validation of DEGs by qRT-PCR

**Figure 4:**
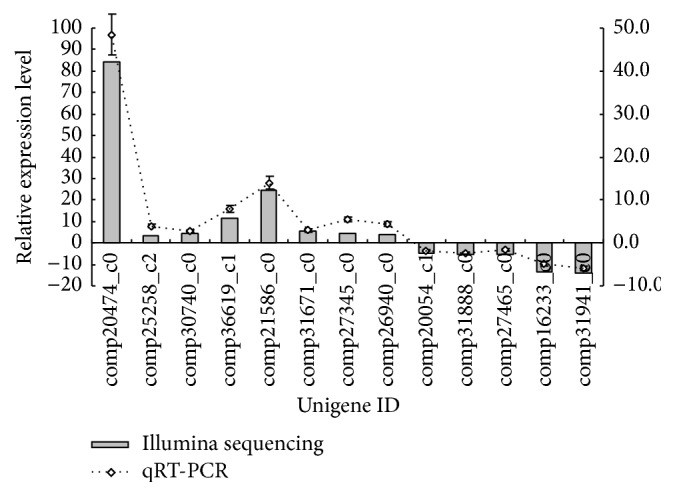
Validation of the gene expression results obtained from Illumina sequencing by qRT-PCR. The left and right vertical ordinates indicate normalized gene expression determined by RNA sequencing and qRT-PCR, respectively.

To confirm the gene expression profiles obtained through Illumina sequencing, the expression patterns of 13 candidate genes (Table S1) were further analyzed with qRT-PCR. For the CH samples, the values are presented as the fold change in gene expression, normalized to the reference gene (actin), relative to the CK samples. Overall, eight genes were upregulated, whereas five genes were downregulated in the CH sample (Figure 4). Although the fold changes in gene expression detected by qRT-PCR were smaller than those detected by Illumina sequencing, the qRT-PCR analysis validated the trend obtained through Illumina sequencing.

### 3.5. Pathway Enrichment Analysis of DEGs

The RM-affected biological pathways were evaluated by enrichment analysis of DEGs. A total of 97 pathways in ramie were probably affected by RM infestation (Table S4). Thirteen pathways were found to be significantly affected by DEGs (*P* < 0.05): four through upregulated DEGs, five by downregulated DEGs, and four enriched by both up- and downregulated DEGs (Table 4). The most significantly affected pathway in ramie following RM infestation was ribosome (*P* < 0.0001), which included 84 upregulated and two downregulated DEGs. The second most significantly affected pathway was that of alpha-linolenic acid (ALA) metabolism (*P* < 0.0001), for which five DEGs were upregulated (Figure S4). Finally, genes involved in photosynthesis and photosynthesis-antenna proteins pathways were also found to be significantly affected by RM infestation, with 30 of these genes being downregulated.

## 4. Discussion

### 4.1. *De Novo* Assembly of the Ramie Transcriptome

Genomewide gene expression profiles can help elucidate the molecular interactions between pests and hosts. In this study, transcriptome changes were analyzed with Illumina sequencing, in order to investigate the defense mechanisms of ramie (the medium-resistant Chuanzhu 8 hybrid) to RM. This resulted in two libraries corresponding to ramie leaves that were RM-infested and uninfested.* De novo* assembly yielded 46,533 unigenes with an average length of 845 bp. In 2013, the whole-plant transcriptome of ramie Zhongzhu 1 was* de novo* assembled, using data yielded from Illumina sequencing. A total of 43,990 unigenes with an average length of 824 bp were obtained from approximately 53 million reads [1]. Subsequently, the transcriptome of this ramie was reassembled by adding new data from a ramie-*Pratylenchus coffeae* interaction study. In that study, a total of 50,486 unigenes with an average length of 853 bp were obtained [23]. More recently, in order to uncover the molecular mechanism behind ramie fiber biosynthesis and development, the transcriptomes of ramie bast, phloem, and xylem were sequenced, and genes involved in cellulose synthesis were studied [24, 25]. Our study focused on insect-induced transcriptome changes in ramie. The resulting data add to the available genetic resources for exploring the developmental biology and defense mechanisms of ramie in response to various stresses.

### 4.2. RM Infestation Induced Transcriptome Changes of Ramie

Recently, there have been studies on changes in ramie transcriptome in response to biotic stresses from* P. coffeae* [23] and* Colletotrichum gloeosporioides* [26], or from abiotic stresses such as drought [27], or cadmium addition [28]. These studies provided insights into the molecular basis of ramie tolerance and resistance to stress. However, transcriptome-related information of ramie-RM interactions is still lacking up to date. In our study, by comparing transcriptome profiling of RM-challenged and unchallenged ramie leaves, 1,980 genes were identified as DEGs, of which 750 were upregulated and 1,230 were downregulated. By searching against a public database, 657 upregulated and 1,142 downregulated genes were functionally annotated. Furthermore, the expression patterns of 13 candidate genes, including three TFs genes, nine defense related protein genes, and one tubulin gene, were confirmed using qRT-PCR. Upon RM infestation, more genes in the ramie leaves were found to be downregulated than upregulated, which is similar to observations in* B. vulgaris*-*Plutella xylostella* interactions [14].

### 4.3. Transcription Factor Responding to RM

A transcriptional control of stress, or responsive gene expression, by TFs is crucial in a plant's response to various biotic and abiotic stresses [29, 30]. Well-known TFs involved in plant defense responses to pathogens or pest attacks include WRKY, ERF, MYB, bZIP, and NAC [30–33]. TFs can activate plant defense responses or be involved directly in different defense signaling pathways [34–36].* WRKY3* and* WRKY6* of tobacco are two insect-responsive WRKY genes; they directly regulate the accumulation of jasmonate (JA) and trypsin proteinase inhibitors and indirectly regulate JA signaling-mediated defenses. Silencing of these genes resulted in increased susceptibility to herbivores, indicating that* WRKY3* and* WRKY6* play important roles in the herbivore resistance of plants [37].* OsERF3*, an ERF type TF of rice, was found to mediate resistance to the striped stem borer, mostly likely by suppressing MAPK repressors and modulating JA, SA, ethylene, and H_2_O_2_ pathways [38].* Arabidopsis* transcription factor* AtMYB12* can regulate a number of pathways (including the phenylpropanoid pathway) in transgenic tobacco. This results in an increased accumulation of rutin, and enhanced resistance to* Spodoptera litura* and* Helicoverpa armigera* [39]. Furthermore,* AtMYB44* can regulate resistance of* Arabidopsis* to the green peach aphid and diamondback moth by activating EIN2-affected defenses [40]. Previously, comparative transcriptome analysis revealed that, for ramie, drought stress and root lesion nematode infection regulated 24 TF genes (containing three NACs, two MYBs, and one EFR) and 40 TFs genes (containing 10 bHLHs, five MYBs, two NACs, and one ERF), respectively [20, 27]. In this study, the expression of 87 TFs was affected by RM larvae infestation (29 were upregulated and 58 were downregulated) and most of them belonged to bHLH (19), EFR (14), ZFP (14), MYB (8), NAC (6), and WRKY (4) families. Overall, we assume that the insect defense mechanisms that involve these TFs are complicated.

### 4.4. Protease Inhibitors Responding to RM

Insects feed directly on plant tissue, and the plant endogenous enzyme inhibitors obstruct this digestion by influencing the digestive amylases and proteases present in the insect guts [41]. Transgenic research showed that some endogenous protease inhibitors (PIs) of plants displayed antiherbivore characteristics. For example, bean *α*-amylase inhibitor 1 gives transgenic cowpeas an increased resistance to two storage pests,* Callosobruchus maculatus* and* C. chinensis, *which cause severe damage to cowpea seeds during storage [42]. Furthermore, the barley trypsin inhibitor (*CMe*) increases the resistance of transgenic rice to* Sitophilus oryzae *[43], and trypsin inhibitors from other plants have shown to increase resistance to insects [44–48]. In our study, 14 genes encoding for different protease inhibitors were found to respond to RM larvae infestation. These 14 genes included trypsin inhibitors (comp15649_c0, comp27345_c0, comp30853_c0, and comp38186_c0), alpha-amylase/subtilisin inhibitors (comp28610_c0 and comp29690_c0), polygalacturonase inhibitors (comp24833_c0 and comp27076_c0), and a xyloglucanase inhibitor (comp36158_c1). Three of these genes (comp15649_c0, comp39830_c0, and comp36636_c0) were also annotated by GO analysis for involvement in the insect defense response in the biological process group. Additionally, several genes were upregulated following RM infestation. This included four genes encoding trypsin inhibitors, such as comp15649_c0 (upregulated 18.6-fold), comp27345_c0 (4.7-fold), comp30853_c0 (4.4-fold), and comp38186_c0 (7.3-fold), as well as two alpha-amylase/subtilisin inhibitor encoding genes, being comp28610_c0 (8.7-fold) and comp29690_c0 (2.6-fold). These results imply that these genes play active roles in ramie-pest defense.

### 4.5. Antioxidant Enzymes Responding to RM

Plants respond to many forms of biotic stress by generating reactive oxygen species (ROS) that participate in defensive signaling and potentiate a hypersensitive response (HR) at the infection site [49]. Immediately after tissue damage caused by herbivore feeding, plants transiently produce ROS (such as the superoxide anion) in the damaged tissue. They additionally produce H_2_O_2_ both locally and systemically throughout the plant [50]. After RM infestation, gene expression of two lignin-forming anionic peroxidases (comp30740_c0 and comp32955_c0), two peroxidases (comp24676_c0 and comp33647_c0), one glutathione peroxidase (comp36311_c0), and one catalase (comp191149_c0) was upregulated. Furthermore, gene expressions of two peroxidases (comp30251_c0 and comp31888_c0) were downregulated. Peroxidase (POD), catalase (CAT), and glutathione peroxidase (GPX) are well-known oxidative stress-related proteins that participate in ROS metabolism [51, 52]. POD is important in ROS generation, whereas CAT and GPX are important in ROS scavenging [52, 53]. A number of studies suggested that POD and CAT play important roles in plant resistance to herbivory [54, 55]. These enzymes were also reported to be involved in ramie resistance to nematodes and anthracnose fungi [26, 56]. In addition, studies also showed that GPX increases plant tolerance to different stress types and regulates cellular immune responses [57, 58]. Moderate amounts of ROS are beneficial, but increased levels of ROS can inhibit cell migration and proliferation. It can even cause severe tissue damage. Therefore, cells must develop strategies for the detoxification of these molecules [59]. In general, plants appear to balance generating ROS as a defensive mechanism and producing ROS-detoxifying enzymes to cope with their own oxidative damage [60]. Hence, oxidative stress-related genes are not uniformly regulated (some upregulated and others downregulated) in leaf tissue in response to insect infestation [61].

### 4.6. ALA Metabolism Influenced by RM Infestation

ALA metabolism is one of the pathways that was significantly affected by RM infestation; the end products of this pathway are (−)-methyl-jasmonate (MeJA) or (+)-7-isomethyl-jasmonate. Upregulation of five genes in this pathway leads to accumulation of JA. JA accumulation usually triggers the biosynthesis of phytoalexins and affects the expression of PR genes synergistically, or by antagonizing the action of other plant hormones [62]. In higher plants, JA is synthesized mainly via the octadecanoid pathway, and lipoxygenase (LOX) is a key enzyme that oxidizes alpha-linolenic acid in the first step of this pathway [63]. In RM-infested ramie, two potential LOX encoding genes (comp29491_c1 and comp29491_c2) were found to be upregulated, and two genes (comp37093_c0 and comp25237_c0) were downregulated. The octadecanoid pathway, however, was not significantly affected. Additionally, the efficiency of the JA mediated defense response varied in different herbivore-plant interaction systems and either decreased [64] or increased resistance [37]. Therefore, the role of JA and JA regulation in ramie-RM interactions needs to be investigated in future studies.

## 5. Conclusions

In summary, to our knowledge, this is the first study on transcriptome changes in ramie leaves induced by insects (RM larvae). The transcriptomes of RM-infested and uninfested ramie leaves were sequenced using Illumina sequencing, and 29,988 and 26,759 unigenes were obtained, respectively. Comparative transcriptome analysis showed that the expression of 1980 genes changed substantially in ramie leaves in response to infestation with RM larvae. Differential expression of some genes was confirmed by qRT-PCR. Many genes encoding for TFs, PIs, and antioxidant enzymes, which are thought to be involved in insect resistance, were regulated by RM larvae infestation. Further characterization of the targets of these genes will help us understand the details of ramie insect resistance.

## Supplementary Material

The supplementary materials contain 8 files, they are some important data related to the methods and results of the presented study. These data make paper easier to read and understand.

## Figures and Tables

**Table 1:** Summary of sequencing and assembly results.

	CK	CH	Total
Total clean reads	40,187,616	62,784,364	
Total clean nucleotides (nt)	4,149,401,262	6,340,667,793	
GC percentage (%)	51.29	50.73	
*N* percentage (%)	0.04	0.04	
Q20 percentage (%)	90.39	90.35	
*Contigs*			
Total number	2,704,882	4,239,322	3,582,696
Total length (nt)	129,385,682	185,382,731	208,724,373
N50 length (nt)	47	43	49
Mean length (nt)	48	44	58
*Transcripts*			
Total number	52,165	58,093	75,553
Total length (nt)	66,692,367	72,503,398	88,128,254
N50 length (nt)	1,846	1,849	1933
Mean length (nt)	1,278	1,248	1166
*Unigenes*			
Total number	26,759	19,988	46,533
Total length (nt)	27,761,126	29,962,009	39,341,510
N50 length (nt)	1,708	1,678	1,585
Mean length (nt)	1,037	982	845

**Table 2:** Number of unigenes annotated in five public databases.

Database	Annotated number	300 ≤ length < 1000	Length ≥ 1000
Nr	24,217	8,771	11,840
Swiss-Prot	17,948	6,299	9,198
GO	20,736	7,115	10,781
COG	9,089	2,608	5,394
KEGG	5,929	2,008	2,914
Total	**24,327**	8,808	11,842

**Table 3:** Summary of differentially expressed genes annotated as transcription factors.

Gene family	Upregulated	Downregulated	DEG number
AP2	1	0	1
ARF	1	1	2
bHLH	5	14	19
bZip	1	0	1
C2H2	0	2	2
CAMTA	0	1	1
COL	0	1	1
EFR	5	9	14
GATA	0	2	2
GLK	0	1	1
TRY	1	0	1
HSF	1	1	2
HY5	0	1	1
MYB	3	5	8
NAC	5	1	6
ORG	0	1	1
RF2	0	3	3
TGA3	0	1	1
TT2	0	1	1
WRKY	2	2	4
YABBY	0	1	1
ZFP	4	10	14
Total	29	58	87

**Table 4:** List of pathways significantly enriched in differentially expressed genes (*P* < 0.05).

Pathway term	DEGs tested	*P* value	Pathway ID
*Pathways for both upregulated and downregulated DEGs*			
Ribosome	84 (23.46%)	9.13*E* − 11	ko03010
Glycosphingolipid biosynthesis-globo series	4 (1.12%)	4.27*E* − 03	ko00603
Zeatin biosynthesis	6 (1.68%)	5.95*E* − 03	ko00908
Ubiquinone and other terpenoid-quinone biosyntheses	7 (1.96%)	1.43*E* − 02	ko00130
*Pathways for upregulated DEGs*			
Alpha-linolenic acid metabolism	5 (1.4%)	6.95*E* − 04	ko00592
Valine, leucine, and isoleucine degradation	13 (3.63%)	7.32*E* − 03	ko00280
Arachidonic acid metabolism	4 (1.12%)	2.43*E* − 02	ko00590
Isoquinoline alkaloid biosynthesis	4 (1.12%)	4.74*E* − 02	ko00950
*Pathways for downregulated DEGs*			
Carotenoid biosynthesis	8 (2.23%)	1.95*E* − 03	ko00906
Photosynthesis	21 (5.87%)	2.43*E* − 03	ko00195
Photosynthesis: antenna proteins	9 (2.51%)	2.32*E* − 02	ko00196
Sulfur metabolism	7 (1.96%)	3.06*E* − 02	ko00920
Pentose and glucuronate interconversions	10 (2.79%)	3.73*E* − 02	ko00040
