# Supplementary material for: Transcriptome Analysis of Ramie (Boehmeria nivea L. Gaud.) in Response to Ramie Moth (Cocytodes coerulea Guenée) Infestation
Source: Biomed Res Int. 2016 Feb 29;2016:3702789. doi: 10.1155/2016/3702789 (PMC4789370; doi:10.1155/2016/3702789)
Supplement: Supplementary file 1 — The supplementary materials contain 8 files, they are some important data related to the methods and results of the presented study. These data make paper easier to read and understand. [file 3702789.f1.zip › Supplementary materials/Fig. S1-Length distribution of unigenes in CK, CH and Integrated CK+CH libraries..doc]

Figure S1 Length distribution of assembled unigene of three libraries: CK, CH and CH+CK. The result showed similar trends of length distribution in three libraries.
