# Supplementary material for: Transcriptome Analysis of Ramie (Boehmeria nivea L. Gaud.) in Response to Ramie Moth (Cocytodes coerulea Guenée) Infestation
Source: Biomed Res Int. 2016 Feb 29;2016:3702789. doi: 10.1155/2016/3702789 (PMC4789370; doi:10.1155/2016/3702789)
Supplement: Supplementary file 1 — The supplementary materials contain 8 files, they are some important data related to the methods and results of the presented study. These data make paper easier to read and understand. [file 3702789.f1.zip › Supplementary materials/Fig. S2-Gene Ontology (GO) classifications of assembled unigenes..doc]

Figure 2 Gene Ontology classifications of assembled unigenes. The x- axis indicated the sub- categories of the three main functional categories: cellular component, molecular function and biological process. The left y- axis indicates the percentage of a specific category of unigenes in that main category. The right y-axis indicates the number of unigenes in each subcategory.
