# Supplementary material for: Transcriptome Analysis of Ramie (Boehmeria nivea L. Gaud.) in Response to Ramie Moth (Cocytodes coerulea Guenée) Infestation
Source: Biomed Res Int. 2016 Feb 29;2016:3702789. doi: 10.1155/2016/3702789 (PMC4789370; doi:10.1155/2016/3702789)
Supplement: Supplementary file 1 — The supplementary materials contain 8 files, they are some important data related to the methods and results of the presented study. These data make paper easier to read and understand. [file 3702789.f1.zip › Supplementary materials/Fig. S3-Histogram presentation of clusters of orthologous groups (COG) classification..doc]

Figure S3

Figure S3 Histogram presentation of clusters of orthologous groups (COG) classification. Out of 24,217 Nr hits, 9,089 unigenes were assigned to 25 COG classifications.
