# Supplementary material for: Transcriptome Analysis of Ramie (Boehmeria nivea L. Gaud.) in Response to Ramie Moth (Cocytodes coerulea Guenée) Infestation
Source: Biomed Res Int. 2016 Feb 29;2016:3702789. doi: 10.1155/2016/3702789 (PMC4789370; doi:10.1155/2016/3702789)
Supplement: Supplementary file 1 — The supplementary materials contain 8 files, they are some important data related to the methods and results of the presented study. These data make paper easier to read and understand. [file 3702789.f1.zip › Supplementary materials/Fig. S4-Up-regulated genes involved in alpha-linolenic acid metabolism that were affected by ramie moth infestation..doc]

**Figure S4**

Up-regulated genes (in red boxes) involved in alpha-linolenic acid metabolism affected by ramie moth infestation. 3.1.1.4: comp38966_c0, 4.2.1.92: comp38024_c0, OPCL1: comp29721_c0, ACX: comp37093_c0 and comp38139_c0.
