# Supplementary material for: Transcriptome Analysis of Ramie (Boehmeria nivea L. Gaud.) in Response to Ramie Moth (Cocytodes coerulea Guenée) Infestation
Source: Biomed Res Int. 2016 Feb 29;2016:3702789. doi: 10.1155/2016/3702789 (PMC4789370; doi:10.1155/2016/3702789)
Supplement: Supplementary file 1 — The supplementary materials contain 8 files, they are some important data related to the methods and results of the presented study. These data make paper easier to read and understand. [file 3702789.f1.zip › Supplementary materials/Table S1-Primer sequences used for qRT-PCR..doc]

**Table S1 Primers sequences used for the qRT-PCR.**

| unigene ID | Description | primer sequence (5'-3') | log2(FC) |
| --- | --- | --- | --- |
| comp20474_c0 | Polyphenol oxidase | F: TCGTTGCCAGCCGAGTAG | 6.4 |
|  |  | R: CGCCAGATGATTTGTTATG |  |
| comp31063_c0 | LoxC-like | F: CGGTTTCGAGGGAGGATG | 4.7 |
|  |  | R: TGCGAGAACGGCTTTAGG |  |
| comp21586_c0 | Tubulin alpha-2 chain | F: CGCTGAACGACGACTGAA | 4.6 |
|  |  | R: GGTCTCGCTGAAGAAGGTG |  |
| comp36619_c1 | Class I chitinase | F: AACACCGGCGATGAAGCT | 3.6 |
|  |  | R: CCTAACGAAACAATAACCC |  |
| comp31671_c0 | NAC transcription factor 053 | F: TCGACGAAGAACCGGAAT | 2.4 |
|  |  | R: TTACTCTACAAATGACCCAC |  |
| comp27345_c0 | Trypsin proteinase inhibitor | F: TGACCCAAACCCTAACCC | 2.2 |
|  |  | R: GGCAAGAAGCAGCGAAAC |  |
| comp26940_c0 | WRKY transcription factor 17 | F: CGCTTGTAGGCGTAGTGG | 1.9 |
|  |  | R: GGCGGATTCTTGGGATTG |  |
| comp25258_c2 | Polyphenol oxidase | F: TGTACCGCCAGATGGTCATT | 1.9 |
|  |  | R: GGGCATTCTTGGGTTTCC |  |
| comp30740_c0 | Lignin-forming anionic peroxidase | F: CCGCCTCTTGCTCACTTAC | 1.2 |
|  |  | R: CATTCGCCTCCATTTCCA |  |
| comp20054_c1 | Putative disease resistance RPP13-like protein 1 | F: ACAGAACGCAATAGACCC | -2.3 |
|  |  | R: GACGACACGCAGCGAAAT |  |
| comp31888_c0 | Peroxidase 63 | F: AGTCTCGTTCCTGGCGTA | -2.4 |
|  |  | R: CACTGCTCGGAGTTCACC |  |
| comp27465_c0 | Ethylene-responsive transcription factor | F: GGGCGTGAGGAAGAGGAAGTG | -2.5 |
|  |  | R: GTCGTAGGAGCCGAGCCAGAT |  |
| comp16233_c0 | Pathogenesis related protein 1 isoform 1 | F: TTCCCTCACATTCCAGTAG | -3.7 |
|  |  | R: CGTGCCCGTTGGCGTATT |  |
| Internal control | ramie actin gene | F: AGTGGAGAAGCGTGGTTT | 0 |
|  |  | R: ATGAATGACGATAATGGC |  |
